# Effect of Surface Cleaning and Heat Treatment on Shear Bond Strength of Cement-Contaminated Zirconia

**DOI:** 10.1055/s-0045-1811225

**Published:** 2025-08-22

**Authors:** Laksika Suwannachot, Niyom Thamrongananskul, Suparaksa Yamockul, Atikom Surintanasarn

**Affiliations:** 1 Esthetic Restorative and Implant Dentistry Program, Faculty of Dentistry, Chulalongkorn University, Pathum Wan, Bangkok, Thailand; 2 Department of Prosthodontics, Faculty of Dentistry, Chulalongkorn University, Pathum Wan, Bangkok, Thailand

**Keywords:** re-cementation, resin cement, contamination, shear bond strength, surface treatment, zirconia

## Abstract

**Objective:**

This study aimed to evaluate the shear bond strength (SBS) of zirconia to dual-cured resin cement following various cleaning protocols and surface treatments after cement contamination.

**Materials and Methods:**

Seventy-seven zirconia specimens (5 × 5 × 2.5 mm ^3^ ) were fabricated, polished, and randomly divided into seven groups ( *n* = 11). Ten specimens per group were tested for SBS, and one was reserved for scanning electron microscopy. Group C− was cemented without surface treatment, while group C+ received air abrasion prior to cementation. Groups F − , F + , FT + , S − , and S+ were initially air-abraded and then contaminated with resin cement. F-series groups were cleaned using a furnace, and S-series with a piezoelectric scaler. Among these, F + , FT + , and S+ received additional air abrasion prior to re-cementation, whereas F− and S− did not. Groups F+ and S+ were re-cemented immediately, whereas FT+ was re-cemented 7 days after surface re-treatment. All specimens were artificially aged before SBS testing.

**Statistical Analysis:**

One-way ANOVA followed by Tukey's HSD post-hoc test was used for statistical analysis (α = 0.05).

**Results:**

Statistically significant differences in SBS were found among the groups ( *p* < 0.05). Group F+ demonstrated the highest bond strength (6.60 ± 1.10 MPa), followed by groups C+ (6.59 ± 0.94 MPa) and S+ (5.43 ± 0.87 MPa), with no significant differences among these three. The lowest SBS was observed in group C− (2.66 ± 0.51 MPa). Intermediate values were recorded in groups F− (4.17 ± 0.96 MPa), FT+ (4.41 ± 0.95 MPa), and S− (4.60 ± 1.17 MPa), which did not differ significantly from each other.

**Conclusion:**

Cleaning cement-contaminated zirconia using a furnace or piezoelectric scaler, followed by air abrasion and immediate re-cementation, significantly improves SBS to levels comparable with clean, air-abraded zirconia. Air abrasion enhances bonding efficacy, while delayed re-cementation may diminish bond strength even after surface re-treatment.

## Introduction

Zirconia-based ceramics have gained widespread popularity in dental restorations due to their excellent mechanical properties, aesthetic quality, and biocompatibility. 1 2 3 Resin cement is commonly used to bond zirconia crowns to teeth or implant abutments, with clinical studies reporting survival rates as high as 93.5% over 5 years. 4 5 However, achieving durable adhesion between zirconia and resin cement remains challenging. Inadequate surface treatment can lead to debonding, with studies reporting failure rates of up to 15% over 5 years due to improper surface preparation. 6 Similarly, a 1.3% debonding rate has been observed in implant-supported zirconia crowns, primarily due to loss of retention. 7

When an indirect restoration debonds without damage to the restoration or abutment, re-cementation is often preferred due to its time and cost efficiency. 8 9 However, residual cement remaining on the intaglio surface may compromise fit, retention, and bond strength. This can increase the risk of secondary caries or periodontal inflammation, ultimately affecting the long-term success of the restoration. 10 11 Palacios et al 12 reported that residual cement remained on 46% of the internal surface of zirconia copings, significantly impairing bond strength. Moreover, mixed failure modes have been observed when self-adhesive resin cements are used. 13 14

Effective cleaning of the zirconia surface is therefore essential before re-cementation. 15 16 Various methods have been proposed for cement removal, including bur abrasion, chemical agents, heat treatment, and airborne-particle abrasion using aluminum oxide (Al _2_ O _3_ ). 17 Heat treatment, often used in laboratory settings, involves heating the restoration to weaken or thermally degrade residual cement, facilitating its removal without damaging the zirconia. 18 In routine laboratory procedures involving implant-supported zirconia crowns, intentional debonding using heat treatment is occasionally performed. This may be done to reuse an existing abutment or to adjust and correct the zirconia crown prior to final placement. This approach is considered conservative, as it preserves the original crown. However, zirconia is sensitive to high temperatures; exposure above 1,170°C can induce irreversible phase transformation from the tetragonal to the monoclinic phase, leading to surface roughening, microcracks, and reduced mechanical properties. 1 2 19

In clinical setting, heat treatment is not commonly used due to the lack of access to furnaces, requiring transportation of the restoration to a laboratory. Delays between surface treatment and re-cementation may expose the zirconia to environmental factors such as air and humidity, which can reduce surface energy and compromise bonding efficacy. 20 21 In contrast, immediate re-cementation following surface treatment helps preserve surface integrity and bonding potential. Previous studies have shown that immediate air abrasion prior to bonding reduces the risk of contamination and enhances adhesion. 15 20 21 22 23

An alternative method for cement removal is the use of a piezoelectric scaler. These devices are clinically convenient and have been shown to remove residual cement effectively without causing significant damage to the zirconia surface. 24 25 26 Unlike heat treatment, piezoelectric scaling does not carry the risk of thermal degradation, making it a safer option for chairside use. However, limited data are available regarding its influence on bond strength following re-cementation.

In addition to cleaning, surface preparation plays a critical role in bonding to zirconia. Airborne-particle abrasion is widely accepted as a method to increase surface roughness and micromechanical retention, thereby improving bond strength and contributing to long-term clinical success. 27 28 However, the effects of a second air abrasion following cleaning—particularly in the context of re-cementation—remain underexplored.

Therefore, the aim of this study was to evaluate the shear bond strength (SBS) of zirconia to dual-cured resin cement following different surface cleaning and preparation protocols. The null hypothesis was that there would be no significant difference in bond strength among the various treatment approaches.

## Materials and Methods

The required sample size was determined using G*Power 3.1 software (Heinrich Heine University, Düsseldorf, Germany), based on mean and standard deviation values of SBS from a pilot study. An α level of 0.05 and a statistical power of 0.90 were applied, resulting in a calculated effect size of 0.631. This calculation indicated that a minimum of eight specimens per group was necessary. To account for potential specimen loss and to include one specimen per group for scanning electron microscopy (SEM) analysis, the sample size was increased to 11 specimens per group (10 for SBS testing and 1 for SEM analysis), yielding a total of 77 specimens for the study.

Seventy-seven zirconia blocks were sectioned from the pre-sintered zirconia discs (KATANA, Kuraray Noritake, Tokyo, Japan) using a diamond precision saw (Isomet, Low Speed Saw, Isomet Precision Saw, Buehler, Illinois) under constant water cooling. The initial block dimensions were 6.2 × 6.2 × 3.1 mm ^3^ , to compensate for sintering shrinkage. Specimens were sintered according to the manufacturer's instructions, then adjusted to final dimensions of 5 × 5 × 2.5 mm ^3^ . The unbonded side of each block was marked as a dot at the corner using a high-speed round diamond bur. Samples were cleaned in distilled water using an ultrasonic bath for 5 minutes and air-dried before surface treatment.

All zirconia blocks were randomly allocated into seven groups ( *n* = 11) according to the number of cementations, cement removal method, and surface treatment ( Table 1 ). For groups not requiring furnace cleaning (C-, C + , S-, and S + ), the specimens were embedded in epoxy resin using double-sided tape (3M ESPE, Sumare, Brazil) to protect the bonding surface. Each ceramic block was then positioned in the center of a cylindrical polyvinyl chloride (PVC) mold and filled with epoxy resin. Once the epoxy resin had fully set, the tape was removed from the ceramic samples. The specimens were then cleaned in distilled water using an ultrasonic bath for 10 minutes to remove any residual adhesive. However, groups requiring furnace decontamination (F-, F + , FT + ) were embedded in the resin block after the cement removal process.

**Table 1:** Summary of cleaning methods and surface treatments used in each experimental group

Experimental group	Symbol	Number of cementation procedures	First cementation	Cleaning method	Second cementation	Storage time before cementation	Description
Negative control	C−	1	No air abrasion	–	–	–	Initial cementation without surface treatment
Positive control	C+	1	Air abrasion with alumina particles	–	–	–	Initial cementation with alumina air abrasion
Furnace	F−	2	Air abrasion with alumina particles	Heat treatment	No air abrasion	–	• Air abrasion before initial cementation Heat treatment at 500°C for 5 minutes before re-cementation
Furnace + air abrasion	F+	2	Air abrasion with alumina particles	Heat treatment	Air abrasion with alumina particles	–	• Air abrasion before initial cementation Heat treatment at 500°C for 5 minutes Air abrasion before re-cementation
Furnace + air abrasion + 7-day delay	FT+	2	Air abrasion with alumina particles	Heat treatment	Air abrasion with alumina particles	7 days	• Air abrasion before initial cementation Heat treatment at 500°C for 5 minutes Air abrasion Storage for 7 days before re-cementation
Ultrasonic scaler	S−	2	Air abrasion with alumina particles	Piezoelectric scaler	No air abrasion	–	• Air abrasion before initial cementation Cleaning with piezoelectric scaler (power level 12)
Ultrasonic scaler + air abrasion	S+	2	Air abrasion with alumina particles	Piezoelectric scaler	Air abrasion with alumina particles	–	• Air abrasion before initial cementation Cleaning with piezoelectric scaler (power level 12) Air abrasion before re-cementation

All bonding surfaces were polished using wet silicon carbide abrasive paper (#100, #360, #600) at 300 rpm for 20 seconds each. Group C− served as a negative control with no surface treatment prior to cementation. Group C+ was treated with air abrasion using 50 μm Al _2_ O _3_ particles (Cobra, Renfert GmbH, Hilzingen, Germany) at 0.25 MPa using a laboratory air abrasion unit (Basic Sandblaster, Renfert GmbH, Hilzingen, Germany), from a distance of 10 mm for 20 seconds. The remaining groups (F-, F + , FT + , S − , S + ) underwent the same surface treatment as group C+ and were contaminated with resin cement, followed by different methods of cement removal and surface treatment before repeating the cementation. The surface treatment involved air abrasion for 20 seconds at a 10-mm distance, using a pressure of 2.5 bars. A thin layer of self-adhesive resin cement (RelyX U200 automix, 3M ESPE, Seefeld, Germany) was then applied to the surfaces of the specimens. A Mylar strip was placed over the uncured cement, and pressed with 5 N for 60 seconds using a 10 mm ball indenter (OD100, Odeme Dental Research, Luzerna, SC, Brazil). Excess cement was removed with a microbrush. The specimens were then light-cured for 40 seconds using a light-emitting diode (LED) unit (Demi Plus, Kerr Corp, Orange, California, United States), with the light tip positioned directly on the Mylar strip at 1,200 mW/cm ^2^ . All specimens were stored in water for 24 hours.

For groups F − , F + , and FT + , the specimens were placed in a furnace (Programat 700/G2, Ivoclar Vivadent) to remove cement. The firing parameters were: closing time ( *S* ) = 06:00, temperature increase ( *t* ) = 50°C, holding temperature ( *T* ) = 500°C, holding timer ( *H* ) = 05:00, vacuum on temperature ( *V* 1) = 200°C, vacuum off temperature (V2) = 499°C, and cool-down gradient (L) = 0. These parameters were adapted from a previous study. 18 After the heat treatment for cement decontamination, the specimens were left to cool for 10 minutes. Afterward, residual cement was removed using a steam cleaner (Evolution Steam Cleaner, Silfradent Srl, Forlì, Italy) at 8.0 kg/cm ^2^ until it became invisible. Finally, the specimens were cleaned in an ultrasonic bath with distilled water for 10 minutes and dried. F− received no additional surface treatment, whereas F+ and FT+ were air-abraded under previously described conditions. FT+ was stored for 7 days before re-cementation.

Groups S− and S+ were cleaned using a piezoelectric scaler (Acteon Satelec P5 Newtron XS, Acteon Group, Mérignac, France) set to scaling mode at power level 12. Manual cleaning was performed along both the *x* and − *x* axes, with the scaler tip No.1 at a 15° angle to the surface until residual cement was no longer visible. Specimens were then cleaned in an ultrasonic bath with distilled water for 10 minutes and dried. S− received no additional surface treatment, while S+ underwent air abrasion before re-cementation.

Bonding procedures were performed on all specimens using self-adhesive dual-cured resin cement (RelyX U200 automix, 3M ESPE, Seefeld, Germany) following the manufacturer's instructions. Bonding was conducted at 25°C by a single operator. Resin cement was injected into transparent custom-made silicone molds to form standardized posts (2.38 mm diameter × 2.00 mm height). Excess cement was removed with a microbrush, then light-cured with an LED unit at 1,200 mW/cm ^2^ for 40 seconds. Post dimensions were confirmed with a digital caliper.

Specimens were stored in distilled water in a laboratory incubator at 37°C for 24 hours, then subjected to 5,000 thermocycles between 5 and 55°C with a 30-second dwell time and 2-second transfer time to simulate 6 months of oral aging.

After thermocycling, specimens were performed SBS testing using a universal testing machine (EZ-SX, Shimadzu, Kyoto, Japan) with chisel blade at 1 mm/min crosshead speed until failure. Results were recorded in MPa. A stereomicroscope (SZ 61, Olympus Corporation, Tokyo, Japan) at 40× magnification was used to examine failure modes. Samples with visible defects were discarded and replaced. Failure types were classified using the Adhesive Remnant Index (ARI) ( Table 2 ). 29

**Table 2:** Description of Adhesive Remnant Index (ARI)

ARI	Criteria
1	100% of the resin remained on the zirconia surface
2	90% of the resin remained on the zirconia surface
3	10–89% of the resin remained on the zirconia surface
4	Less than 10% of the resin remained on the zirconia surface
5	No resin remained on the zirconia surface

One additional specimen of each group was produced, performing all kinds of surface treatment before cementation (C− and C + ) and re-cementation (F − , F + , FT + , S-, and S + ). It was then dried in a laboratory desiccator and coated with a layer of gold before being examined under an SEM (JSM-IT700HR, JEOL, Tokyo, Japan) at different magnification (100× to 15,000 × ) to characterize surface morphology.

**Fig. 1:**
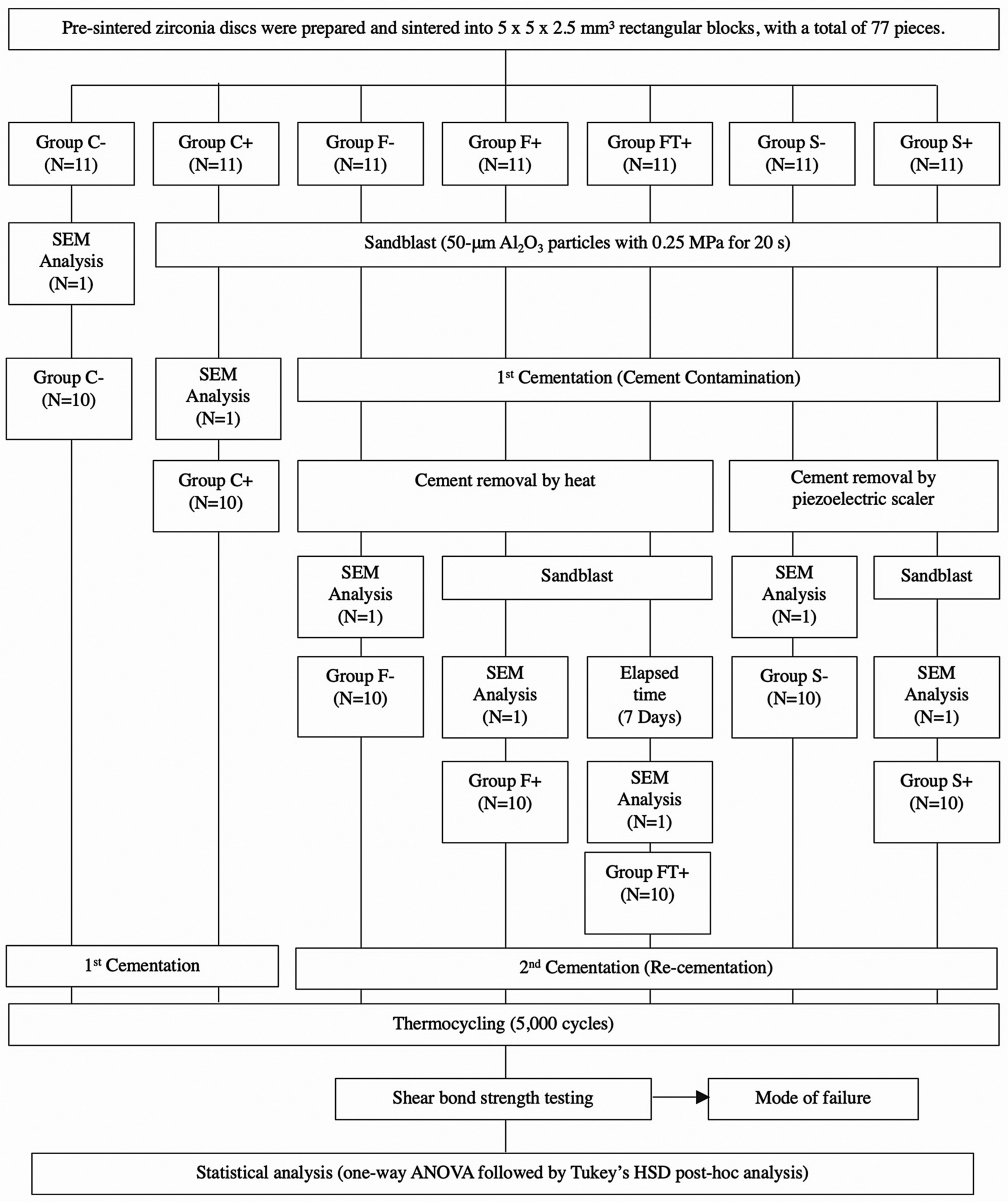
Flowchart of the experimental procedure.

The SBS values (MPa) of all specimens were analyzed using SPSS (program version v29.0.1). Normality was assessed using the Kolmogorov–Smirnov test, and homogeneity of variance with Levene's test. One-way ANOVA and Tukey's HSD post-hoc test were used to compare SBS among groups (α = 0.05). A flowchart of the experimental procedure is shown in Fig. 1 .

## Results

All specimens survived thermocycling for 5,000 cycles without premature debonding, confirming their suitability for SBS testing. The mean SBS values (in MPa) and standard deviations are presented in Table 3 .

**Table 3:** Descriptive statistics for shear bond strength (MPa)

Group	Cleaning method and surface preparation	*n*	Mean ± SD (MPa)
C−	No cement contamination, no air abrasion	10	2.66 ± 0.51 ^C^
C+	No cement contamination, air abrasion	10	6.59 ± 0.94 ^A^
F−	Cement contamination, furnace only	10	4.17 ± 0.95 ^B^
F+	Cement contamination, furnace + air abrasion	10	6.60 ± 1.10 ^A^
FT+	Cement contamination, furnace + air abrasion, 7-day delay	10	4.41 ± 0.96 ^B^
S−	Cement contamination, piezoelectric scaler only	10	4.60 ± 1.17 ^B^
S+	Cement contamination, piezoelectric scaler + air abrasion	10	5.43 ± 0.87 ^AB^

Abbreviation: SD, standard deviation.

Note: Groups with different superscript letters are statistically different ( *p* < 0.05).

Group C− exhibited the lowest bond strength (2.66 ± 0.51 MPa), while group C+ demonstrated significantly higher SBS (6.59 ± 0.94 MPa). Among the cement-contaminated groups, F+ exhibited the highest SBS (6.60 ± 1.10 MPa), which was significantly greater than that of F− (4.17 ± 0.95 MPa), FT+ (4.41 ± 0.96 MPa), and S− (4.60 ± 1.17 MPa) ( *p* < 0.05). Notably, F+ and S+ (5.43 ± 0.87 MPa) showed SBS values statistically comparable to the positive control (C + ), while all other groups demonstrated significantly lower values. Delayed re-cementation following air abrasion led to a significant reduction in bond strength, as evidenced by the difference between F+ (6.60 ± 1.10 MPa) and FT+ (4.41 ± 0.96 MPa).

**Fig. 2:**
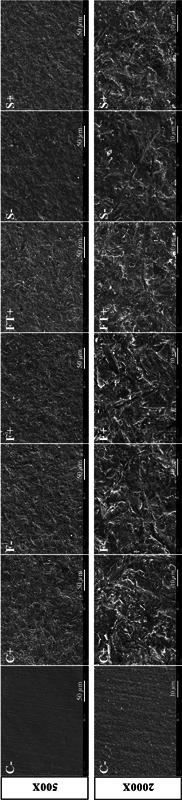
Representative scanning electron microscopy (SEM) images of each group at 500× and 2,000× magnification, showing surface characteristics after treatment.

SEM analysis ( Fig. 2 ) revealed distinct differences in surface morphology. Group C− showed only minor polishing marks, while all groups subjected to air abrasion (C + , F − , F + , FT + , S − , and S + ) displayed visibly roughened zirconia surfaces. Among these, groups F+ and S + , which received a second round of air abrasion after cement removal, exhibited deeper and more defined surface grooves than their counterparts without post-cleaning air abrasion.

Failure mode analysis indicated that the majority of specimens exhibited adhesive failures, corresponding to an ARI score of 5. A few specimens in groups F+ and FT+ demonstrated mixed failure modes. One specimen in each of these groups retained residual cement on the zirconia surface, corresponding to an ARI score of 4, indicating less than 10% cement remained on the bonding interface.

## Discussion

The clinical success of zirconia restorations is predominantly influenced by the bond strength achieved following cementation. 30 This study assessed the SBS of zirconia subjected to different cleaning methods and surface treatments prior to cementation with dual-cured resin cement. The null hypothesis proposed that there would be no significant difference in SBS between the experimental groups and either the positive or negative control groups. Based on the results, the null hypothesis is rejected.

The findings showed that surface cleaning and treatment after cement contamination could significantly influence bonding outcomes. Notably, two methods—furnace cleaning followed by air abrasion and immediate re-cementation (F + ), and piezoelectric scaler cleaning followed by air abrasion and immediate re-cementation (S + )—demonstrated SBS values statistically comparable to the positive control group (C + ), which represented initial cementation. These groups also showed significantly higher SBS values than the untreated negative control (C − ).

In particular, the combination of heat treatment, air abrasion, and immediate re-cementation produced SBS values statistically indistinguishable from those of the positive control. Previous studies have reported that thermal degradation of resin cement is an effective cleaning method that can restore bonding potential close to initial cementation. 17 18 31 In this study, a maximum temperature of 500°C for 5 minutes was used, based on prior findings indicating that dual-cured resin cement disintegrates at ∼363 ± 71°C. 18 Although some manufacturers suggest higher temperatures (e.g., 550°C for 15 minutes), this study adopted a conservative approach to avoid structural damage to zirconia. Past studies have shown that temperatures exceeding 1,170°C can cause irreversible phase transformations, resulting in microcracking and reduced mechanical strength. 1 2 19 The thermally degraded cement appeared as a flaky, opaque layer, which was easily removed with steam cleaning and ethanol wiping.

In routine laboratory procedures involving implant-supported zirconia crowns, intentional debonding using heat treatment is occasionally performed. This may be done to reuse an existing abutment or to adjust and correct the zirconia crown prior to final placement. This approach is considered conservative, as it preserves the original crown. While furnace-based cleaning was effective in this study, it is not a realistic option in most clinical settings due to the absence of furnaces in typical dental offices. The process typically requires outsourcing to a laboratory, resulting in added cost and treatment delay. In contrast, piezoelectric scalers are widely accessible and commonly used in clinical practice, making them a more cost-effective and time-efficient alternative for chairside application. In practice, dislodged crowns found in the clinic are usually unheated and must be sent to an external laboratory for thermal treatment, which can delay the clinical workflow. To reflect this real-world scenario, the present study included a group that did not undergo furnace treatment, thereby mimicking a more clinically relevant situation.

The use of a piezoelectric scaler followed by air-particle abrasion proved to be a clinically practical and effective cleaning method. This protocol produced SBS values comparable to those of the furnace-treated group and the positive control. SEM analysis confirmed that these surfaces were free from residual cement and exhibited microtexture patterns consistent with effective micromechanical retention. Because piezoelectric scalers are routinely used in dental practices, this method offers a highly accessible alternative. Previous research has shown that piezoelectric scaling does not compromise the mechanical properties of zirconia when used to remove excess cement or dental calculus. 24 25 26

Air abrasion with 50 µm alumina particles at 0.25 MPa significantly improved SBS when compared with untreated zirconia. Moreover, performing a second round of air abrasion after cement removal further increased SBS values across all cleaning groups. SEM images at 2,000× magnification revealed no microcracks in any air-abraded specimens. Previous studies have shown that air abrasion at optimal pressures (0.20–0.35 MPa) and distances (1–20 mm) does not adversely affect zirconia's biaxial flexural strength and may even enhance it. 32 33 34 Although some findings suggest that microcracks may form during alumina abrasion, 35 these effects appear to be mitigated once the restoration is bonded with resin cement. 36 These results support the theory that air abrasion improves resin–zirconia bonding by increasing micromechanical interlocking through surface roughening. 15 27 28 37 38

A reduction in SBS was observed in the FT+ group, where re-cementation was delayed after air abrasion. This decline is likely attributable to surface contamination or a decrease in surface energy over time. Previous studies have similarly reported reduced bond strength with delayed bonding due to diminished surface free energy. 20 21 39

Failure mode analysis showed that all groups successfully underwent 5,000 thermocycles, representing 6 months of simulated intraoral function, 40 41 with no pre-test debonding. Adhesive failure (ARI score of 5) was the most frequently observed mode across all groups. Occasional mixed failures were found in groups F+ and FT + , which may be attributed to stress concentration at the cement–zirconia interface. This phenomenon is recognized as a common limitation of SBS testing. 42 43 44

This study focused exclusively on mechanical surface cleaning methods and did not evaluate the use of chemical cleaning agents, including universal or zirconia-specific cleaning solutions. Moreover, the *in vitro* design, limited thermocycling protocol (5,000 cycles), absence of mechanical loading, and use of a single resin cement formulation may limit the clinical generalizability of the findings. Future studies should investigate the comparative effectiveness of chemical and mechanical cleaning strategies, and incorporate extended aging, fatigue loading, and multiple resin cements to better reflect real-world conditions.

Within the scope of this *in vitro* study, the results demonstrate that appropriate cleaning and surface treatment protocols can restore SBS in cement-contaminated zirconia to levels comparable with initial cementation. Although only micromechanical aspects of bonding were assessed, the findings suggest promising strategies for clinical application. Future studies should evaluate the long-term durability of re-cemented zirconia restorations and the interaction of cleaning techniques with different types of resin cement formulations under extended intraoral simulation.

## Conclusion

Within the limitations of this *in vitro* study, both furnace and piezoelectric scaler cleaning, when followed by air abrasion and immediate re-cementation, effectively restored the bond strength of cement-contaminated zirconia to levels comparable with initial cementation. A second air abrasion step further enhanced micromechanical retention, whereas delayed re-cementation led to a notable reduction in bond strength. These findings support the clinical application of accessible and effective cleaning protocols, offering practical guidance for managing debonded zirconia restorations in both chairside and laboratory settings.
